# A Longitudinal Study of Foreign Language Enjoyment and L2 Grit: A Latent Growth Curve Modeling

**DOI:** 10.3389/fpsyg.2021.720326

**Published:** 2021-08-30

**Authors:** Majid Elahi Shirvan, Tahereh Taherian, Mojdeh Shahnama, Elham Yazdanmehr

**Affiliations:** ^1^Department of Foreign Languages, University of Bojnord, Bojnord, Iran; ^2^Department of English Language and Literature, Yazd University, Yazd, Iran; ^3^Department of English Language, Ferdowsi University of Mashhad, Mashhad, Iran; ^4^Department of English Language, Attar Institute of Higher Education, Mashhad, Iran

**Keywords:** foreign language enjoyment, L2 grit, latent growth curve model, initial level, growth level, longitudinal study

## Abstract

In line with the dynamic shift in SLA domain and the need for the development of suitable methods to explore the dynamics of emerging concepts in the field such as grit and enjoyment, in the present research, we intended to investigate the growth of foreign language enjoyment (FLE) and L2 grit over time. To do this, we used a bivariate latent growth curve model (LGCM) to examine the covariance between 437 EFL learners' initial and growth levels of L2 grit and FLE in four measurement occasions of 2 week intervals. The data were collected *via* the original foreign language enjoyment scale and the L2 grit scale. The model including the covariance between intercepts and slopes of FLE and L2 grit was tested *via* Mplus 7. The findings indicated an increasing trend in the association between the growth levels of both variables. That is, the means of both L2 grit and FLE were larger at their growth level than their initial level. Also, analyzing the co-variations in the model showed that the covariances between the intercepts and slopes of FLE and L2 grit were statistically significant. This would point to the existence of a parallel process (co-development) of FLE and L2 grit. This result also implied that an increase in the level of FLE among the participants was strongly correlated with an increase in the level of L2 grit during the whole course. The findings were discussed with reference to previous studies in the literature and the implications were also provided.

## Introduction

Recently, as a result of the advent of positive psychology (PP) in SLA (MacIntyre and Mercer, 2014; MacIntyre et al., 2016) and the ever-increasing interest in the complexity and complex dynamic systems theory (CDST), examining the dynamics of PP variables has emerged as a new line of research. L2 grit and foreign language enjoyment (FLE) are among such variables. Grit refers to individuals' tendency to express perseverance and passion for long-term goals (Duckworth et al., 2007) while FLE pertains to the degree of perceiving L2 learning pleasant in class by students (Dewaele and MacIntyre, 2014).

In the light of dynamic systems in classroom learning, several factors may interact to affect FLE such as classroom environment (Dewaele and MacIntyre, 2014; Dewaele et al., 2018; Elahi Shirvan and Taherian, 2020) as well as teacher and learner variables (Dewaele and MacIntyre, 2014; Dewaele et al., 2018, 2019a; Jiang and Dewaele, 2019; Elahi Shirvan et al., 2020). The existing literature has also introduced grit as a predicting factor for educational success (Datu et al., 2017), which can be related to positive emotions (Datu et al., 2016, 2018), well-being criteria such as life satisfaction (Datu et al., 2016; Vainio and Daukantaite, 2016), and mental well-being (Vainio and Daukantaite, 2016). In fact, these previous studies indicated that grit can predict optimal emotions which can provide insights into the association between L2 grit and FLE. More specifically, it has been argued that there are more chances that grittier individuals make positive attributions, have a more positive growth mindset which in turn results in developing further positive emotions (Duckworth et al., 2009; Hill et al., 2016).

Given the plausible association between L2 grit and FLE as two PP variables in SLA, the longitudinal dynamic association, consistent with the CDST line of research, between these two variables has not been investigated yet. Wei et al. (2019) contended that when individual difference (ID) variables are used alongside each other, they may grow together and have mediating effects on one another, and by doing so, they can affect the quality of language learning. Furthermore, regarding the rational underlying the longitudinal examination of the covariance between L2 grit and FLE as two individual difference (ID) variables, it should be noted that (Dörnyei and Ryan, 2015, p. 6) raised serious issues concerning the classic four assumptions of IDs, one of which is that (b) “IDs are relatively *stable*.” They contended that “when we look more closely, individual learner characteristics are not stable but show salient temporal variation….” (p. 6). Quite similar to this argument, Ellis and Larsen-Freeman (2006, p. 563) maintained that “To attribute causality to any one variable (or even a constellation of variables) without taking time into account is misguided.”

Dörnyei and Ryan (2015) also maintained that:

Reframing individual differences as complex dynamic systems has the potential to mitigate many of the failings associated with the classic ID conceptualization……. Arguably the most significant contribution of a complex dynamic systems approach is in its role as an overriding guiding principle that positions *change* rather than stability as the norm, moving us away from static conceptualizations of learners toward embracing notions of change and growth (p. 6)

Given the above explanations, examining the longitudinal interplay between the L2 grit and FLE can provide us with deeper conceptualization of their temporal covariance as well as the occasional change and stability of these variables which is not taken into account in single-shot data collection (Larsen-Freeman, 2016). Therefore, there is a need for CDST compatible models (see Hiver and Al-Hoorie, 2019; Hiver et al., in press) to take into account the interactive effects of these variables over time. To access more accurate and detailed information about the relative changes between L2 grit and FLE over time, in this study we used a longitudinal growth curve model (LGCM). The flexibility of a LGCM shifts the focus toward within-individual changes and more accurate and precise interpretations concerning the association between L2 grit and FLE.

Via the use of LGCM as CDS compatible method, we followed three main objectives in the present research. First, we intended to describe the change in grit and FLE through time for the present participants. Conceptually, this was achieved by forming a regression line (a growth curve) plotting both variables over time for all participants of the study. Second, we aimed to achieve an average intercept, as initial level, as well as an average slope, as rate of change, for all individuals of the study for two latent variables of FLE and L2 grit. Finally, we examined the covariance between two latent variables of L2 grit and FLE to understand the co-development of these two variables over time.

## Review of Related Literature

Inspired by Fredrickson's (2001) broaden-and-build theory, MacIntyre and Gregerse (2012) introduced PP in SLA. They contended that positive affective factors can help language learners with absorbing further language input (Dewaele et al., 2019b). Under the influence of Pekrun's control-value theory of achievement emotion (Pekrun, 2006) as a second inspiring theory for researching emotions in SLA, positive emotions like enjoyment are seen emerging from the learning activities. Li (2021) maintained that the two influential theories of pp in SLA, namely, Fredrickson (2001) and Pekrun (2006) acknowledge the pivotal role of positive emotions in students' well-being and performance.

## Foreign Language Enjoyment

Apart from anxiety, little attention was paid to other emotions in second language acquisition (SLA) until 2012. However, the emergence of PP shifted the focus from anxiety to more diverse L2 learning emotions. Therefore, three phases of emotions have been introduced in the field of SLA, moving from the “Emotion Avoidance Phase” to “Anxiety-Prevailing Phase” to a final phase called “Positive and Negative Emotions Phase” which as its name implies incorporates positive and negative emotions both and their complex dynamic interaction (Dewaele and Chengchen, 2020). This third phase was influenced by the introduction of PP in SLA in 2010 (MacIntyre and Gregerse, 2012).

Positive psychologists do not deny the existence or impact of negative emotions (Dewaele and Li, 2018) but rather than focusing on defining problems and overcoming learner deficiencies, they attempt to enhance the positive emotion by encouraging greater engagement as well as increasing the appreciation of meaning in life and its activities (MacIntyre and Mercer, 2014). This new perspective has been particularly welcomed in SLA as the long-standing great focus on what has wrongly led to the relative neglect of the effect of positive emotions on language learning for years (Dewaele and MacIntyre, 2014, 2016, 2019; Dewaele and Dewaele, 2017).

One major PP theory is the Broaden-and-Build theory (Fredrickson, 2001). In terms of the Broaden-and-Build Theory (Fredrickson, 1998, 2013), positive emotions help to expand a learner's thought-action repertoire and form the psychological, physical, intellectual, and social resources necessary for his/her present and prospective development. Therefore, expressing positive emotions in learning a foreign language can enhance a learner's consciousness of language input as well as perceptions of linguistic features, and can increase their application of different problem-solving techniques (Piechurska-Kuciel, 2017; Boudreau et al., 2018), which lead to an expansion of the students' foreign language knowledge-base. In addition, positive emotions can moderate the influence of negative emotions associated with issues of learning foreign languages, enhance resilience from difficulties, and form social links in class by creating active approaches to both teachers and peers (Dewaele and Alfawzan, 2018), which points to the additional value of the turn in SLA domain to positive emotions.

One positive emotion worth investigation is FLE. So far, latent factors of FLE (Dewaele and MacIntyre, 2016; Li et al., 2018) as well as its antecedents and outcomes (Dewaele and Alfawzan, 2018) and its association with language anxiety (Dewaele and MacIntyre, 2014, 2016; Elahi Shirvan and Taherian, 2021) have been explored. Dewaele and MacIntyre (2014) introduced a FLE scale with 21 items that deal with positive emotions about the learning process, peers and teacher. Overall, they found a small negative association (*r* = −0.34) between FLE and foreign language classroom anxiety (FLCA) among 1,740 foreign language students from all over the world. Such results suggest that the two emotions are not in a strong see-saw association, but appear to operate as two different but correlated dimensions of experience. Further quantitative analysis revealed that higher FLE and lower FLCA were associated with age, a high level of multilingualism, mastery in the FL, matching the perceived competence of peers in the FL class, and being in university rather than secondary school settings (Dewaele and MacIntyre, 2014).

With a focus on continuous system change and interconnectedness, several studies have shown that FLE is complex, dynamic, and multifaceted (Dewaele and Dewaele, 2017; Elahi Shirvan and Talebzadeh, 2018b; Pavelescu and Petrić, 2018; Elahi Shirvan et al., 2020; Elahi Shirvan and Taherian, 2021). Following this dynamic perspective, some studies have investigated FLE from a person-centered approach, called idiographic approach (Elahi Shirvan and Talebzadeh, 2018a; Elahi Shirvan and Taherian, 2020; Elahi Shirvan et al., 2020) and from a variable-centered approach, also called nomothetic perspective (Dewaele and Dewaele, 2017; De Ruiter et al., 2019) while some used a mixed methods approach (Dewaele and MacIntyre, 2019).

As the first attempt to investigate dynamics of FLE from a nomothetic perspective, Dewaele and Dewaele's (2017) pseudo-longitudinal study explored the FLE changes through time, following a dynamic approach. They compared three groups of students categorized in terms of age and observed a moderate increase in FLE through time. An additional regression analysis showed that fewer learner-internal and teacher-centered variables managed to predict FLE at both the end and the beginning of secondary education vs. the middle part. These findings suggested that sources of FLE were dynamic and changeable between the age of 12 and 18.

Acknowledging the dynamic nature of emotions, De Ruiter et al. (2019) investigated intra-individual dynamics of FLE that are embedded in the growing context of classroom. Using the technique of Kohonen's Self-Organizing Maps (SOM), they aimed to explore the intra-individual process of FLE and FLA along teachers' emotional support during the interactions between a teacher and his students in terms of two dyadic interactions. The results showed the emergence of repeated patterns of teacher support, student enjoyment and anxiety during these interactions which highlighted the self-organizing quality of these student-teacher dyadic interactions, the bidirectional form of this process, and, generally, the conception of students and teachers as dynamic systems. Besides, the particular quality of the emergent patterns shows that the conventional positive relationship between teacher support and student emotion might be generalized to processes of real-life.

In a recent study, Elahi Shirvan et al. (2020) used a time-based sampling design of ecological momentary assessment in order to explore the dynamism of multiple aspects of FLE indifferent time scales in an intermediate EFL course. Applying open ended interviews during months, journals during the weeks, enjoy meters within minutes as well as the idio dynamic approach within seconds, their findings showed that enjoyment in foreign language changes in terms of a hierarchy of timescales, from moment-to-moment shifts to those from month to month.

Additionally, Dewaele and Dewaele (2020) explored the extent to which FLE varies at a unique point of time in the presence of two different teachers for a single FL. Their participants were a subgroup of 40 students with one primary teacher and a secondary teacher for the same foreign language. Their findings revealed that the participants reported remarkable higher FLE with the primary teacher. They discussed their findings in association with significantly more positive perceptions of the primary teacher and his frequent application of the foreign language in class.

As an innovative method compatible with CDST (see Hiver and Al-Hoorie, 2019), application of LGCM for investigating FLE has been recently used by Elahi Shirvan and Taherian (2020). They employed a LGCM method to trace the growth in 367 undergraduates' FLE along a semester in a general English course. This study revealed that although their participants' FLE increased dramatically throughout the semester, the significance of the intercept and slope variances for FLE pointed to their heterogeneity in FLE along the research time. In addition, the initial level of FLE did not show to predict its growth along the course. Also, very recently, Pan and Zhang (2021), *via* a longitudinal study, explored the fluctuations of FLE in during a 14-week period and their association with learners' motivation and personality traits. They found FLE quite dynamic over time under the influence of both motivational (ideal L2 self, ought-to self) and personality trait factors such as extroversion.

## L2 Grit

Grit, a recently-introduced personality trait which is a positive, non-cognitive skill, is shown to be promising in life success as well as academic success (McCain, 2017). It has been defined along two dimensions of perseverance of effort (PE) and consistency of interest (CI) (Duckworth et al., 2007). Both dimensions of grit contribute significantly to success as perseverance of effort facilitates the achievement of mastery in spite of failure and consistency of interest is a key to deliberate practice to gain mastery (Credé et al., 2016).

Many investigations have attempted to reveal how grit relates to academic and non-academic achievement. These investigations revealed that grit is positively correlated with non-academic outcomes including retention in military programs as well as achievement in the National Spelling Bee competition (e.g., Duckworth et al., 2007; Duckworth and Quinn, 2009; Eskreis-Winkler et al., 2014) and retention in career and married life (Eskreis-Winkler et al., 2014). Besides, grit has shown to be positively associated with academic achievement and certain constructs including classroom management (Banse and Palacios, 2018), teacher's support (Keegan, 2017; Lee and Drajati, 2019; Lee and Hsieh, 2019; Lee, 2020; Yoon et al., 2020), academic achievement (Akos and Kretchmar, 2017), self-efficacy (Usher et al., 2019), engagement (Von Culin et al., 2014; Wolters and Hussain, 2015; Aparicio et al., 2017; Hodge et al., 2017; Fosnacht et al., 2018; Wei et al., 2019), education motivation (Piña-Watson et al., 2015), and achievement goals (Chen et al., 2018).

In the SLA domain, the early works of research used the general grit scale to measure language learners' grit. To begin with, Lake (2013) investigated the correlation between grit and a number of PP variables. He surveyed 800 female Japanese university students who learned English language *via* a new edition of Duckworth et al.'s (2007) general scale and showed that grittier students enjoyed a stronger interest in spending time and putting efforts in L2 learning. Changlek and Palanukulwong (2015) recruited 183 Thai students to explore gritty students' motivational features. They found that high-achievers were grittier and grit was positively associated with students' motivation and negatively with anxiety.

In a similar vein, Wei et al. (2019) observed a positive low association between grit and English language scores of 832 secondary school students in China. Yamashita (2018) estimated no correlation between 78 Japanese learners' grit and GPA. Interestingly, the PE dimension of grit showed a negative correlation with GPA for a sub-sample of the population. It is noteworthy that these studies all employed the general grit scales introduced by Duckworth and colleagues (Duckworth et al., 2007; Duckworth and Quinn, 2009) and assessed learners' grit globally and not locally and not embedded in the context of L2 learning.

These inconclusive results raise the point that an L2-specific grit scale was needed to act as a more accurate measurement of achievement in an L2 setting (Sudina et al., 2020). Consequently, Teimouri et al. (2020) constructed the L2 grit scale to assess the domain-specific grit in SLA. These results based on a sample of 191 EFL learners, showed higher correlations between the L2-grit scale and L2 learners' outcome than the Grit–O, as evidenced by large attenuation-corrected correlations for the L2 grit.

What is common among the existing literature is that the L2 grit works of research are not only limited in size, but they have scarcely addressed its dynamic nature and its relationship with other emotions experienced in the context of SLA.

## L2 Grit and L2 Emotions

As mentioned before, Teimouri et al. (2020) constructed a domain-specific grit scale to measure learners' passion and perseverance in learning a second language. These researchers explored how the general grit scale and the domain-specific grit scale dealt with various emotional, motivational, and language-related effects. Their results revealed a positive correlation between L2-specific grit and enjoyment, attention, intended effort, growth mindset, willingness to communicate, and other language achievement measures. Besides, a negative correlation was found with language anxiety. In this research, the correlations between L2-specific grit and L2 achievement variables were negligible. Also, only PE showed to be correlated with L2 outcome measures for both L2 specific and general grit.

Lee (2020) looked into the role of classroom enjoyment and grit in L2 willingness to communicate in primary school, high school, and university students. The results showed that both variables were significant positive antecedents of L2 willingness to communicate in the three groups. Recently, Feng and Papi (2020) reported that perseverance of effort (POE), and not consistency of interest (COI), should be regarded as a positive predictor of L2 persistence. Finally, Wei et al. (2019) observed that grit is associated with foreign language performance overtly and covertly *via* foreign language enjoyment. They also found a mediating role of enjoyment on grit. They concluded that those who are grittier have better performances and exhibit better self-reactions and positive emotions including FLE. Moreover, classroom environment affected the correlation of grit and enjoyment in such a way that in a positive environment, students increased their effort and by doing so enjoyed the learning. Despite the recent interest in the exploration of L2 grit and emotions, especially positive ones, in SLA, the dynamic interaction of L2 grit and positive emotions like FLE still needs to be explored from a longitudinal perspective *via* the use of suitable methods. One of the methods is LGCM.

## LGCM

Researchers in a variety of disciplines have used LGCM in a structural equation modeling (SEM) framework to describe and analyze changes in individual's attributes, including personal characteristics, behaviors, and education outcomes over time. LGCM addresses important methodological concerns for analyzing panel data over the traditional regression methods and mean comparisons. Firstly, the dynamic quality of these models views the repeated measure as a process that unfolds over time not as a status at two distinct time points (Coyne and Downey, 1991). Secondly, when an individual change has a non-linear trajectory, LGCM techniques are appropriate to show complexities of such change (Willett and Sayer, 1994). Thirdly, concerning the growing quality of the change process, LGCM techniques provide empirical researchers with a richer and wider range of research questions; those dealing with the nature of individual development (Willett, 1988). Fourthly, such analysis provides a thorough understanding of the dynamic association between different time-dependent cognitive and non-cognitive factors than conventional analytical methods.

Considering the recent research on the dynamics of emotions in SLA (e.g., Gkonou et al., 2017; Boudreau et al., 2018), and the link between positive emotions and grit (Datu et al., 2018), it can be hypothesized that L2 learners' growth in their L2 grit can be influenced by a change in their level of FLE during a foreign language course. Using a LGCM, we firstly aimed to trace the variations of two time-varying non-cognitive factors, FLE and L2 grit through time for each subject in the study. As a second objective, the current study aimed at obtaining an average intercept, or initial level, as well as an average slope, or the rate of change, for all individuals, each with its own variance for two latent variables of FLE and L2 grit. Finally, we analyzed the dynamic association or covariance between FLE and L2 grit to explore the co-development of these two variables over time.

Therefore, the present research was conducted to answer the following questions:

- In what direction and to what extent do English as a foreign language learners' FLE and L2 grit change throughout the semester?
- Do the initial levels of FLE and L2 grit predict their rate of change?
- Is change over time in FLE related to change over time in L2 grit?

### Participants and Setting

The target population was a sample of 437 English as a foreign language (EFL)learners (278 females and 159 males) which were selected from private language institutes of three big cities in Iran. Their level of language proficiency ranged between lower-intermediate to upper-intermediate, according to Oxford Placement Test, and their age ranged from 17 to 34 years. All participants' first language was Persian. They learned English as a foreign language (EFL). The data collection occurred in summer 2020. During this period all the English language courses were held in-person.

### Instrumentation

#### FLE Scale

To measure L2 learners' FLE, we used the Persian version of the original 21-itemscale of FLE (Dewaele and MacIntyre, 2014) developed by Elahi Shirvan et al. (2021). The items of the scale reflected the social dimension of FLE with 9 items (e.g., “We form a tight group,” “There is a good atmosphere”) and the private dimension of FLE in class with 12 items (e.g., “I enjoy it”, “I'm a worthy member of the English class”). Answers to the statements were provided on a 5-point Likert scale (1 = strongly disagree, 2 = disagree, 3 = undecided, 4 = agree, 5 = strongly agree) for the L2 classes. All items were positive statements. The internal consistency of the original scale, as reported by Dewaele and MacIntyre (2014), estimated *via* Cronbach's alpha, was interpreted as high (*r* = 0.86). A one-sample Kolmogorov-Smirnov test attested to the normality of distribution (z = 2.01, *p* = 0.09). Also, Resnik and Dewaele (2020) analyzed and reported the internal consistency of original FLE scale as high (Cronbach's alpha = 0.843, *N* = 12). To measure the reliability of the Persian version of FLE scale, Elahi Shirvan et al. (2021) ran Cronbach's α to examine its internal consistency and McDonald's ω (McDonald, 1985, 1999) to test the composite reliability, which were reported to be 0.88 and 0.89, respectively. The longitudinal confirmatory factor analysis of the scale was also supported by Elahi Shirvan et al. (2021) with partial strong invariance.

#### L2 Grit Scale

To measure L2 grit, we used the scale developed by Teimouri et al. (2020) with two interrelated sub-constructs: consistency of interest and perseverance of efforts in learning a language. The former assesses the changes of students' interest during L2 learning and the latter measures the extent to which learners are persistent in achieving their L2 goals. After a series of statistical analyses (i.e., item analysis, reliability analysis and principal component analysis), Teimouri et al. (2020) retained 12 items, six for consistency of interest (e.g., “My interests in learning English changes from year to year”) and six for perseverance of efforts (e.g., “I will not give up learning English until I master it”). Further analysis showed that the item-total correlations of 3 items did not reach the minimum criterion of 0.40 and were, thus, discarded. The 9 remaining items were then used to constitute the L2 grit scale. They also reported a higher reliability for language-domain-specific grit than domain-general grit (t[190] = 84.34, *p* < 0.001, Cohen's d = 0.81). All the items of the scale were rated on a five-point Likert-type scale ranging from 1 (not at all like me) to 5 (very much like me) *via* the Persian version of the scale provided by Teimouri et al. (2020). The reliability indices of the scale based on Cronbach's α and McDonald's ω were 0.87 and 0.88, respectively.

### Data Collection

The data collection method was questionnaire-based. Both the L2 grit scale and the FLE scale were given to the participants in four measurement occasions with 2-week intervals at the outset of the EFL courses (to take into account the initial status factor). Based on the findings of the previous studies, IDs are susceptible to micro, day to day changes, and macro, weekly and monthly changes. Given the length of a language course, it is contended that the 2-week intervals between the time points in this study could reflect the underlying causal lags in a of course of language learning. This longitudinal data collection helped to measure changes in the target variable through time. It also allowed for estimating the rate of changes at each phase and accounted for inter-individual differences. Questionnaire completion was done step by step in the classroom in the presence of one of the researchers. The participants were made sure of the confidentiality of the information they provided and they all agreed to participate in this research.

### Data Analysis

Prior to the statistical analysis of the collected data, a test of normality was used for all variables. Therefore, *via* kurtosis and skewness estimates and Kolmogorov–Smirnov test of normality, we double-checked the normality of the variables. Also, LGCM was applied to examine the covariances between the initial and growth levels of L2 grit and FLE through the use of multi-wave data (Byrne, 2016) during the four measurement occasions along with the dynamic relationship of the two variables. To do so, the paths from intercept factors to the observed variables of L2 grit and FLE were set as 1. In other words, intercept values were constant across the four measurement occasions for all participants (see Byrne, 2016). Also, following the procedures suggested by Byrne (2016), we constrained the paths from slope factors to the observed variables to 0, 1, 2, and 3. This means that 1, 2, and 3 reflect the same time intervals (4th, 6th, and 8th weeks), with 0 standing for the 2nd week as the starting point of growth of the two variables. For testing the model, we used LGCM in Mplus 7.4 software.

## Results

### Preliminary Analyses

The successful estimation of growth curves relies on the consistency of higher associations of repeated indicators at adjacent occasions than correlations at non-adjacent measurement occasions (Lorenz et al., 2004). As observed in Tables 1, 2, correlation matrix indicated that correlation coefficients between two adjacent occasions (t and t+1) for each factor were higher than correlations between non-adjacent occasions (FLErs ranged from 0.26 to 0.49; L2 grit *rs* ranged from 0.34 to 0.53). Moreover, off-diagonal correlations of the same repeated measure over time (Tables 1, 2) showed to be quite different from each other. These correlations show that a significant slope variation might exist for each global factor trajectory model.

**Table 1:** Correlation matrix between FLEs over time.

	**FLE1**	**FLE2**	**FLE3**	**FLE4**
FLE1	–			
FLE2	0.49	–		
FLE3	0.41	0.47	–	
FLE4	0.26	0.30	0.42	–

*FLE, foreign language enjoyment*.

**Table 2:** Correlation matrix between grits over time.

	**Grit 1**	**Grit 2**	**Grit 3**	**Grit 4**
Grit1	–			
Grit 2	0.53	–		
Grit 3	0.41	0.41	–	
Grit 4	0.36	0.34	0.45	–

*Grit, L2 grit*.

**Figure 1:**
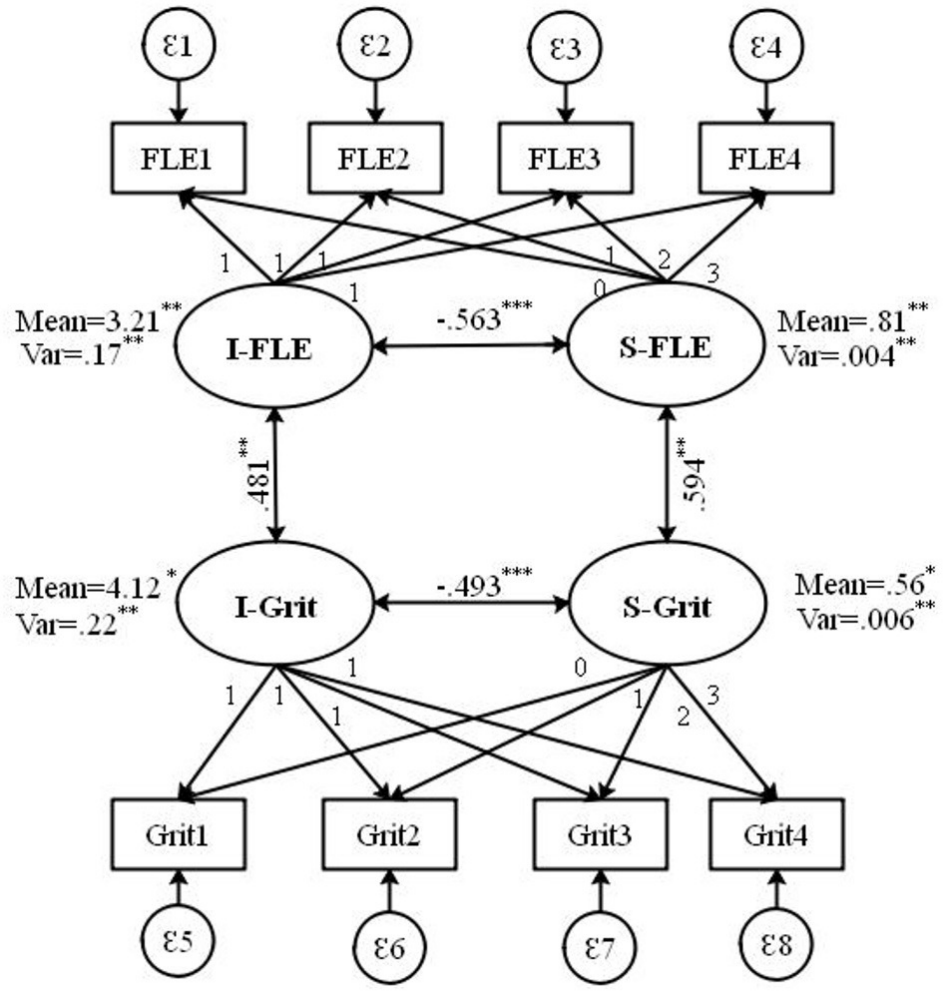
Parallel process model (PPM). I, Initial Level; S, Slope; FLE, foreign language enjoyment; Grit, L2 grit. [χ*2*(*df* ) = 55.324(24), *p* < 0.001; *CFI/TLI* = 0.932/0.923; *RMSEA* (*90% CI*) = 0.055 (0.039, 0.052); *SRMR* = 0.057]. *p < 0.05. **p < 0.01. ***p < 0.001.

*The overall model (see Figure 1) fit indices were acceptable [χ2(df) = 55.324(24), p < 0.001;CFI/TLI = 0.932/0.923; RMSEA (90% CI) = 0.055 (0.039, 0.058); SRMR = 0.057]*.

## Main Analyses

### Direction of Change in FLE and L2 Grit

As for the direction and extent of change in FLE and L2 grit that the participants experienced in the target English course, all parameter means of the two global factors were statistically significant. The results indicated that FLE (*M*_Intercept_ = 3.213, *p* < 0.001; *M*_*Slope*_= 0.814, *p* < 0.001) and L2 grit (*M*_Intercept_ = 4.121, *p* < 0.001; *M*_Slope_ = 0.565, *p* < 0.001) increased across time.

### Heterogeneity in Learners' FLE and L2 Grit

Besides, about the variance of both variables, the findings showed a significant variance in intercept factors of FLE (σ2 = 0. 174, *p* < 0.001) as well as those of L2 grit (σ2 = 0.221, *p* < 0.001) which shows heterogeneity in the participants' FLE and L2 grit in the 1st session of the data collection, the initial session within the model. Moreover, a look at the significance of the slope variances to find the growth patterns of FLE and L2 grit over the semester showed a statistically significant slope variance for FLE (σ2 = 0.004, *p* < 0.001) and L2 grit (σ2 = 0.006, *p* < 0.001). Statistically significant variances for two global factors indicate the presence of inter-individual differences in the FLE and L2 grit developmental patterns. In other words, some participants went through a higher rate of change in FLE and L2 grit, yet some others experienced lower rates of FLE and L2 grit during the course.

### Predictive Power of the Initial States of FLE and L2 Grit

Furthermore, as indicated in Table 3, the negative covariance between the intercept and slope of FLE (*r* = −0.563, *p* < 0.001) and between the intercept and slope of L2 grit (*r* = −0.493, *p* < 0.001) suggested that lower initial scores in learners' FLE and grit exhibited a steeper increase over time. It means that those students who started off at a higher level of FLE and L2 grit exhibited less change in FLE and L2 grit over time and those participants who started off at a lower level of FLE and L2 grit exhibited more change in their FLE and L2 grit over the term.

**Table 3:** Results of the parallel process model (PPM).

	**Intercept growth factors**		**Slope growth factors**		**Correlations among growth factors**				
	**Mean**	**Variance**	**Mean**	**Variance**		**I-FLE**	**I- Grit**	**S-FLE**	**S- Grit**
FLE	3.213^**^	0.174^**^	0.814^**^	0.004^**^	I- FLE	–			
					I- Grit	0.481^**^	–		
Grit	4.121^*^	0.221^**^	0.565^*^	0.006^**^	S-FLE	−0.563^***^	−0.086	–	
					S- Grit	−0.091	−0.493^***^	0.594^**^	–

*I, Initial Level; S, Slope; FLE, foreign language enjoyment; Grit, L2 grit*.

* *p < 0.05*.

** *p < 0.01*.

*** *p < 0.001*.

### Association of Change in FLE and L2 Grit Over Time

The analysis of co-variations in the model showed that the covariance between the intercept factors for the FLE and L2 grit was moderate but statistically significant (0.481, *p* < 0.001). A similar pattern of significant covariance was observed between the FLE and L2 grit slope factors (0.594, *p* < 0.001), which indicated the existence of a parallel process (co-development) between FLE and L2 grit at the global factor level. This result implied that the rise of the participants' FLE was strongly correlated with the rise of their L2 grit during the course though the correlation between participants' FLE and L2 grit was moderate at the beginning of the course.

## Discussion

As the present findings indicated, an increase in enjoyment was associated with the increase in grit over time. This means that the direction of change from initial level to growth levels of these two factors was increasing. Moreover, the mean of growth in both FLE and L2 grit was higher than the mean of their intercepts. This suggests the increase in both variables during the semester. The increase in the intercepts and growth factors of FLE and L2 grit along with the positive association of these two variables can be explained by considering some contextual factors reported in the findings of previous studies, such as teacher's emotional support, teacher's academic support, challenging instruction and assessment, classroom management (e.g., teacher's control, well-organized classroom, challenging and interesting activities), learning engagement, and classroom environment.

In our study, we found a negative covariance of the intercept and slope of FLE (*r* = −0.563, *p* < 0.001) which meant that lower initial scores in learners' FLE demonstrated a steeper increase over time. On the other hand, those participants who started off at a higher level of FLE experienced less change in FLE over time while those learners whose initial level of FLE was very low exhibited more change in their FLE over time. This means that the initial status of FLE (either high or low) does not necessarily guarantee the maintenance of a similarly high or low level of the construct over time. This finding is in agreement with the works of research by Elahi Shirvan and Taherian (2021) and Elahi Shirvan et al. (2021) who concluded that the initial states of FLE did not predict students' growth during the semester. Therefore, we postulate that the high growth of enjoyment in EFL learners with lower initial FLE as well as the inter-individual variance of learners in their initial stage and growth level of enjoyment may be associated with teacher-related factors like teacher's support as speculated in previous studies (e.g., Elahi Shirvan and Taherian, 2021; Elahi Shirvan et al., 2021).

Also, inspired by Fredrickson's Broad-and-Build Theory (2001, 2003) proclaiming that “positive emotions broaden an individual's momentary mindset, and by doing so help to build enduring personal resources' (Fredrickson, 2003, p. 332) and similar to the findings of previous studies (e.g., De Ruiter et al., 2019; Lee, 2020; Elahi Shirvan and Taherian, 2021; Elahi Shirvan et al., 2021), we can conjecture that teachers' emotional support, such as their warmth and friendliness, during the EFL course may increase positive emotions like enjoyment in learners over time. Therefore, it is expected that teacher's warmth and friendliness can create a safe, nurturing environment for students with initially low enjoyment level so that they can experience more enjoyment during later sessions of the course.

The increase in EFL learners' enjoyment during the course might be also possibly supported by the classroom environment, as suggested by the previous studies (e.g., Elahi Shirvan and Taherian, 2020, 2021; Li et al., 2020) helping learners to overcome difficulties in a friendly atmosphere. Also, as contended by Joe et al. (2017), within the safe, positive, and high-caring classroom environment, mutual respects and peer-interactions and social connections among peers are highly encouraged. Within this classroom environment, learning is combined with pleasure and interpersonal relations (Dewaele and MacIntyre, 2014).

Accordingly, in line with the findings of prior works of research (Dewaele and MacIntyre, 2014; Khajavy et al., 2018; Dewaele et al., 2019a; Elahi Shirvan and Taherian, 2021), we can hypothesize that another factor that may influence the growth level of EFL learners' enjoyment can be classroom climate especially social climate that teachers create in classroom. A classroom environment in which students can feel safe to express their opinions might help learners develop a friendly relationship and a social connection with their classmates. This might lead to the increase in EFL learner's confidence and sense of cooperation which in turn might increase the level of enjoyment in the classroom.

Given the increasing pattern in the learners' growth of L2 grit, inspired by the findings of the previous studies (Banse and Palacios, 2018), we can conjecture that the increase in learners' grit during an EFL course may be related to some contextual factors such as teacher's classroom management, which may consist of class control, challenging instruction, tasks and assessment and a well-organized class, teacher's support and classroom engagement.

Banse and Palacios (2018) reported that the level of grit increased in those students who perceived their teacher's high level of care and class management (e.g., control and classroom organization) as a factor to increase their effort in class. High level of classroom management motivates students to be involved with more on-task behaviors in classroom and accordingly increasing their grit. According to Banse and Palacios (2018), a teacher with a well-organized classroom can create an environment for students to divide their long-term goals into smaller ones and try to master the content more deeply. Thus, in terms of classroom management, acceptable classroom management of teachers during an EFL course such as having good control of the situation, creative methods of instruction, encouraging more cooperation among students and a well-organized lesson plan as well as reasonable challenging assessments can be hypothesized to contribute to the increase in EFL learner's perseverance, which in turn may encourage them to make more efforts to improve their performance.

In addition, we can postulate that another possible factor that can increase EFL students' grit may be related to the amount of support that teachers give to their students. Therefore, we can conclude that just like enjoyment, grit can also be considered a teacher-related factor. That is, consistently with the findings of the previous studies (e.g., Keegan, 2017; Lee and Drajati, 2019; Lee and Hsieh, 2019; Lee, 2020; Yoon et al., 2020) we can attribute increase in the growth level of EFL learners' grit to teachers' behavioral and cognitive or meta-cognitive supports such as their encouragement of a sense of cooperation instead of competition, breaking down students' long-term goals into more specific, immediate ones to provide a chance for giving feedback and coaching them to achieve their goals in a more consistent way.

Besides, in alignment with the observations of the previous works of research (e.g., Von Culin et al., 2014; Wolters and Hussain, 2015; Datu et al., 2016; Aparicio et al., 2017; Hodge et al., 2017; Wei et al., 2019) which indicated that those students who are grittier are proved to be more engaged in class activities, engagement can be regarded as another possible factor in the growth of EFL learners' grit.

The present findings revealed that besides a moderate correlation between the participants' FLE and L2 grit at the opening of the course, FLE and L2 grit were co-developed at growth level as well. This means that the increase in enjoyment was significantly associated with the increase in grit during semester and vice versa. One possible reason for the covariance of both variables at the growth level can be related to teacher's support and the contribution of classroom environment to the increase in the growth of both grit and enjoyment. As mentioned previously, in line with the findings of Banse and Palacios (2018), we hypothesized that environmental features of class such as teacher's warmth, care, positive feedback, friendly classmates, and positive class atmosphere can increase learners' confidence and consequently, boost their effort in classroom to improve their performance while enjoying their class. Moreover, Wei et al. (2019) also found the classroom environment as a mediating factor influencing the association between grit and enjoyment. They concluded that a positive classroom environment increased students' grit, which in turn paved the way for an increase in their level of enjoyment. They also found that enjoyment had a mediating role in increasing students' effort through fostering their cognitive and mental resources, as the result of which they were able to improve their foreign language achievement. Therefore, enjoyment and grit are supposed to have reciprocal effects on each other and as the findings of our study showed, this reciprocal influence grows during the semester. This means that when students enjoy the class, they continue to learn and make efforts in learning even if the situation gets difficult and in doing so, their level of enjoyment may increase.

This co-development of grit and enjoyment during an EFL course can also be enhanced by teacher's class management. As highlighted in previous studies (Cameron et al., 2005; Kang, 2005; Elahi Shirvan and Taherian, 2020), we can conjecture that designing well-organized activities with an optimal level of challenge and creating more pair-work and group-work activities can facilitate positive emotions and increase the sense of cooperation and effort among students to reach the desired result (e.g., Duckworth et al., 2009; Hill et al., 2016; Wei et al., 2019). This is consistent with studies which found a positive association between enjoyment and grit. They concluded that grittier students who show better academic behaviors have more optimistic growth mindsets and, therefore, have higher levels of enjoyment.

## Conclusion

We explored the covariance of the initial and growth levels of 437 EFL learners' FLE and grit over four measurement occasions *via* the innovative method of LGCM. The findings indicated a significant and large association between the growth levels of both variables over time. The findings imply that affective engagement in terms of experiencing a positive emotion like enjoyment can be tightly associated with more perseverance and enjoyment for EFL learners during an EFL course. From a pedagogical perspective, the findings indicate that during a language course learners' enjoyment can increase in association with their perseverance and effort. Thus, the initial levels of learners' grit and enjoyment should be regarded by teachers as the benchmark for their evaluation of their learners' states of these variables in a foreign language course. Instead, *via* the activation of contextual stimuli and their pivotal role in the class, as both variables are teacher-related, teachers can direct their learners to the point that they make more efforts and maintain higher level of interest for the long term goal of language acquisition while experiencing more enjoyment during the course than its prior sessions. In other words, with regard to the pedagogical implications of the findings, language teachers should consider grit as a variable within the nomological network of their learners' experience of enjoyment. Thus, given the teacher-dependent nature of enjoyment, any endeavors by language teachers to improve their learners' sense of enjoyment over time can help their learners sustain their efforts and interests for the achievement of English language. On the other hand, due to the bidirectional association of FLE and L2 grit over time, any stimulating behavior by language teachers to encourage further perseverance and consistency of interest in their learners can help them feel more enjoyment in their language learning process. Regarding the limitations of the study, it should be noted that the longitudinal phase of this study was limited to 2-week intervals with the participants filling in the same scales in these intervals. Further research can focus on the temporal covariance of L2 grit and FLE with longer intervals. Also, we should note that the incorporation of qualitative stage could provide us with further explanations regarding our quantitative data analysis. Further studies can consider conducting a mixed-methods study for the interpretation of the findings. Also, the inclusion of the learners' achievement as the outcome as well the predictor in the longitudinal model of this study can provide further details regarding the factors contributing to the co-development of FLE and L2 grit during a foreign language course.

## Data Availability Statement

The raw data supporting the conclusions of this article will be made available by the authors, without undue reservation.

## Ethics Statement

The studies involving human participants were reviewed and approved by University of Bojnord. The patients/participants provided their written informed consent to participate in this study.

## Author Contributions

TT and MS involved in the data collection. All authors have contributed to the development of the idea for research, data analysis, and the process of writing the manuscript.

## Conflict of Interest

The authors declare that the research was conducted in the absence of any commercial or financial relationships that could be construed as a potential conflict of interest.

## Publisher's Note

All claims expressed in this article are solely those of the authors and do not necessarily represent those of their affiliated organizations, or those of the publisher, the editors and the reviewers. Any product that may be evaluated in this article, or claim that may be made by its manufacturer, is not guaranteed or endorsed by the publisher.

## References

[B1] Akos P. Kretchmar J. (2017). Investigating grit at a non-cognitive predictor of college success. Rev. High. Educ. J. Assoc. Stud. High. Educ. 40, 163–186. 10.1353/rhe.2017.0000

[B2] Aparicio M. Bacao F. Oliveira T. (2017). Grit in the path to e-learning success. Comput. Human Behav. 66, 388–389. 10.1016/j.chb.2016.10.009

[B3] Banse H. Palacios N. (2018). Supportive classrooms for Latino English language learners: Grit, ELL status, and the classroom context. J. Educ. Res. 111, 645–656. 10.1080/00220671.2017.1389682

[B4] Boudreau C. MacIntyre P. D. Dewaele J.-M. (2018). Enjoyment and anxiety in second language communication: an idiodynamic approach. Stud. Second Lang. Learn. Teach. 8, 149–170. 10.14746/ssllt.2018.8.1.7

[B5] Byrne B. M. (2016). Structural Equation Modeling with AMOS: Basic concepts, Applications, and Programming, (3rd ed). London: Routledge.

[B6] Cameron C. E. Connor C. M. Morrison F. J. (2005). Effects of variation in teacher organization on classroom functioning. J. School Psychol. 43, 61–85. 10.1016/j.jsp.2007.03.002 19083356

[B7] Changlek A. Palanukulwong T. (2015). Motivation and grit: predictors of language learning achievement. Human. Soc. Sci. 8, 23–36. Available online at: https://he02.tci-thaijo.org/index.php/Veridian-E-Journal/article/view/40089

[B8] Chen C. Ye S. Hangen E. (2018). Predicting achievement goals in the East and West: the role of grit among American and Chinese university students. Educ. Psychol. 38, 820–837. 10.1080/01443410.2018.1458975

[B9] Coyne J. C. Downey G. (1991). Social factors and psychopathology: stress, social support, and coping processes. Annu. Rev. Psychol. 42, 401–425. 10.1146/annurev.ps.42.020191.002153 2018399

[B10] Credé M. Tynan M. C. Harms P. D. (2016). Much ado about grit: a meta-analytic synthesis of the grit literature. J. Pers. Soc. Psychol. 113:492. 10.1037/pspp0000102 27845531

[B11] Datu J. A. D. Valdez J. P. M. King R. B. (2016). Perseverance counts but consistency does not! validating the Short Grit Scale in a collectivist setting. Curr. Psychol. 35, 121–130 10.1007/s12144-015-9374-2

[B12] Datu J. A. D. Yuen M. Chen G. (2017). Grit and determination: a review of literature with implications for theory and research. J. Psychol. Counsell. Sch. 27, 168–176. 10.1017/jgc.2016.2

[B13] Datu J. A. D. Yuen M. Chen G. (2018). The triarchic model of grit is linked to academic success and well-being among Filipino high school students. Sch. Psychol. Q. 33, 428–438. 10.1037/spq0000234 29927277

[B14] De Ruiter N. M. Elahi Shirvan M. Talebzadeh N. (2019). Emotional processes of foreign-language learning situated in real-time teacher support. Ecol. Psychol. 31, 127–145. 10.1080/10407413.2018.1554368

[B15] Dewaele J.-M. Alfawzan M. (2018). Does the effect of enjoyment outweigh that of anxiety in foreign language performance? Stud. Sec. Lang. Learn. Teach. 8, 21–45. 10.14746/ssllt.2018.8.1.2

[B16] Dewaele J.-M. Chen X. Padilla A. M. Lake J. (2019a). The flowering of positive psychology in foreign language teaching and acquisition research. Front. Psychol. 10:2128. 10.3389/fpsyg.2019.02128 31607981 PMC6769100

[B17] Dewaele J.-M. Chengchen L.i (2020). Emotions in second language acquisition: a critical review and research agenda, in A Positive Psychology Perspective on Emotions in SLA, ed Chengchen Li [Special Issue] Foreign Language World [Chinese ??? ] 196, 34–49.

[B18] Dewaele J.-M. Dewaele L. (2017). The dynamic interactions in foreign language classroom anxiety and foreign language enjoyment of pupils aged 12 to 18. A pseudo-longitudinal investigation. J. Euro. Sec. Lang. Assoc. 1, 12–22. 10.22599/jesla.6

[B19] Dewaele J.-M. Li C. (2018). Editorial: Special Issue Emotions in SLA. Stud. Sec. Lang. Learn. Teach. 8, 15–20.

[B20] Dewaele J.-M. MacIntyre P. D. (2014). The two faces of Janus? anxiety and enjoyment in the foreign language classroom. Stud. Second Lang. Learn. Teach. 4, 237–274. 10.14746/ssllt.2014.4.2.5

[B21] Dewaele J.-M. MacIntyre P. D. (2016). Foreign language enjoyment and foreign language classroom anxiety: the right and left feet of the language learner [A], in Positive Psychology in SLA [C]. eds MacIntyre P. D. Gregersen T Mercer S. (Bristol: Multilingual Matters), 215–234.

[B22] Dewaele J.-M. MacIntyre P. D. (2019). The predictive power of multicultural personality traits, learner and teacher variables on Foreign Language Enjoyment and Anxiety, in Evidence-based second language pedagogy: A collection of Instructed Second Language Acquisition studies, eds Sato M. Loewen S. (London: Routledge), 12.

[B23] Dewaele J.-M. Magdalena A. F. Saito K. (2019b). The effect of perception of teacher characteristics on Spanish EFL learners' anxiety and enjoyment. Mod. Lang. J. 103, 412–427. 10.3389/fpsyg.2019.412427

[B24] Dewaele J.-M. Witney J. Saito K. Dewaele L. (2018). Foreign language enjoyment and anxiety in the FL classroom: the effect of teacher and learner variables. Lang. Teach. Res. 22, 676–697. 10.1177/1362168817692161

[B25] Dewaele J. M. Dewaele L. (2020). Are foreign language learners' enjoyment and anxiety specific to the teacher? an investigation into the dynamics of learners' classroom emotions. Stud. Sec. Lang. Learn. Teach. 10, 45–65. 10.14746/ssllt.2020.10.1.3

[B26] Dörnyei Z. Ryan S. (2015). The Psychology of the Language Learner Revisited. New York, NY: Routledge.

[B27] Duckworth A. L. Peterson C. Matthews M. D. Kelly D. R. (2007). Grit: perseverance and passion for long-term goals. J. Pers. Soc. Psychol. 92, 1087–1101. 10.1037/0022-3514.92.6.1087 17547490

[B28] Duckworth A. L. Quinn P. D. (2009). Development and validation of the Short Grit Scale (Grit–S). J. Pers. Assess. 91, 166–174. 10.1080/00223890802634290 19205937

[B29] Duckworth A. L. Quinn P. D. Seligman M. (2009). Positive predictors of teacher effectiveness. J. Positiv. Psychol. 19, 540–547. 10.1080/17439760903157232

[B30] Elahi Shirvan M. Taherian T. (2021). Longitudinal examination of university students' foreign language enjoyment and foreign language classroom anxiety in the course of general English: latent growth curve modeling. Int. J. Bilingual Educ. Bilingual. 24. 10.1080/13670050.2018.1441804

[B31] Elahi Shirvan M. Taherian T. Yazdanmehr E. (2020). The dynamics of foreign language enjoyment: an ecological momentary assessment. Front. Psychol. 11, 1391–1405. 10.3389/fpsyg.2020.01391 32765337 PMC7381172

[B32] Elahi Shirvan M. Taherian T. Yazdanmehr E. (2021). Foreign language enjoyment: a longitudinal confirmatory factor analysis–curve of factors model. J. Multiling. Multicult. Develop. 1–19. 10.1080/01434632.2021.1874392

[B33] Elahi Shirvan M. Talebzadeh N. (2018a). Is transparency an illusion? an idiodynamic assessment of teacher and peers' reading of nonverbal communication cues of foreign language enjoyment. J. Intercult. Commun. Res. 47, 188–206

[B34] Elahi Shirvan M. Talebzadeh N. (2018b). Exploring the fluctuations of foreign language enjoyment in conversation: an idiodynamic perspective. J. Intercult. Commun. Res. 47, 21–37. 10.1080/17475759.2017.1400458

[B35] Elahi Shirvan M. E. Taherian T. (2020). Affordances of the Microsystem of the Classroom for Foreign Language Enjoyment. Human Arenas: Advance online publication.

[B36] Ellis N. C. Larsen-Freeman D. (2006). Language emergence: implications for applied linguistics—Introduction to the special issue. Appl. Linguistics 27, 558–589 10.1093/applin/aml028

[B37] Eskreis-Winkler L. Shulman E. P. Beale S. A. Duckworth A. L. (2014). The grit effect: Predicting retention in the military, the workplace, school and marriage. Front. Psychol. 5:36. 10.3389/fpsyg.2014.00036 24550863 PMC3910317

[B38] Feng L. Papi M. (2020). Persistence in language learning: the role of grit and future self-guides. Learn. Individ. Differ. 81, 1–10. 10.1016/j.lindif.2020.101904

[B39] Fosnacht K. Copridge K. Sarraf S. A. (2018). How valid is grit in the postsecondary context? a construct and concurrent validity analysis. Res. High. Educ. 60, 803–822. 10.1007/s11162-018-9524-0

[B40] Fredrickson B. L. (1998). What good are positive emotions? Rev. General Psychol. 2, 300–319. 10.1037/1089-2680.2.3.300 21850154 PMC3156001

[B41] Fredrickson B. L. (2001). The role of positive emotions in positive psychology: the broaden-and-build theory of positive emotion. Am. Psychol. 56, 218–226. 10.1037/0003-066X.56.3.218 11315248 PMC3122271

[B42] Fredrickson B. L. (2003). The value of positive emotions. Am. Sci. 91, 330–335. 10.1511/2003.4.330

[B43] Fredrickson B. L. (2013). Positive emotions broaden and build. Adv. Exp. Soc. Psychol. 47, 1–53. 10.1016/B978-0-12-407236-7.00001-2

[B44] Gkonou C. Daubney M. Dewaele J.-M. (2017). New insights into language anxiety: theory, research and educational implications. Bristol: Multiling. Matters 9722. 10.21832/9781783097722

[B45] Hill P. L. Burrow A. L. Bronk K. C. (2016). Persevering with positivity and purpose: an examination of purpose commitment and positive affect as predictors of grit. J. Happiness Stud. 17, 257–269. 10.1007/s10902-014-9593-5

[B46] Hiver P. Al-Hoorie A. H Larsen-Freeman D. (in press). Toward a transdisciplinary integration of research purposes methods for complex dynamic systems theory: Beyond the quantitative–qualitative divide. International Review of Applied Linguistics in Language Teaching. Advance online publication.

[B47] Hiver P. Al-Hoorie A. H. (2019). Research Methods for Complexity Theory in Applied Linguistics. London: Multilingual Matters.

[B48] Hodge B. Wright B. Bennett P. (2017). The role of grit in determining engagement and academic outcomes for university students. Res. High. Educ. 59:2. 10.1007/s11162-017-9474-y

[B49] Jiang Y. Dewaele J. M. (2019). How unique is the foreign language classroom enjoyment and anxiety of Chinese EFL learners?. System 82, 13–25. 10.1016/j.system.2019.02.017

[B50] Joe H.-K. Hiver P. Al-Hoorie A. H. (2017). Classroom social climate, self-determined motivation, willingness to communicate, and achievement: a study of structural relationships in instructed second language settings. Learn. Individ. Differ. 53, 133–144. 10.1016/j.lindif.2016.11.005

[B51] Kang S. J. (2005). Dynamic emergence of situational willingness to communicate in a second language. System 33, 277–292. 10.1016/j.system.2004.10.004

[B52] Keegan K. (2017). Identifying and building grit in language learners. English Teaching Forum 55, 2–9. Available online at: https://eric.ed.gov/?id=EJ1156469

[B53] Khajavy G. H. MacIntyre P. D. Barabadi E. (2018). Role of the emotions and classroom environment in willingness to communicate: applying doubly latent multilevel analysis in second language acquisition research. Stud. Sec. Lang. Acquisit. 40, 605–624. 10.1017/S0272263117000304

[B54] Lake J. (2013). Positive L2 self: Linking positive psychology with L2 motivation, in Language Learning Motivation in Japan (Second Language Acquisition), eds Apple M. T. Da Silva D. Fellner T. (Bristol: Multilingual Matters).

[B55] Larsen-Freeman D. (2016). Classroom-oriented research from a complex systems perspective. Stud. Second Lang. Learn. Teach. 6, 377–393. 10.14746/ssllt.2016.6.3.2

[B56] Lee J. S. (2020). The role of grit and classroom enjoyment in EFL learners' willingness to communicate. J. Multiling. Multicult. Develop. 174:6319. 10.1080/01434632.2020.1746319

[B57] Lee J. S. Drajati N. A. (2019). Affective variables and informal digital learning of English: Keys to willingness to communicate in a second language. Austr. J. Educ. Technol. 35, 168–182. 10.14742/ajet.5177

[B58] Lee J. S. Hsieh J. C. (2019). Affective variables and willingness to communicate of EFL learners in In-class, out of class, and digital contexts. System 82, 63–73. 10.1016/j.system.2019.03.002

[B59] Li C. (2021). A Control–Value Theory approach to boredom in English classes among university students in China. Mod. Lang. J. 105, 317–334. 10.1111/modl.12693

[B60] Li C. Dewaele J. M. Jiang G. (2020). The complex relationship between classroom emotions and EFL achievement in China. Appl. Ling. Rev. 11, 485–510. 10.1515/applirev-2018-0043

[B61] Li C. Jiang G. Dewaele J. M. (2018). Understanding Chinese high school students' foreign language enjoyment: validation of the Chinese version of the Foreign Language Enjoyment Scale. System 76, 183–196. 10.1016/j.system.2018.06.004

[B62] Lorenz F. O. Wickrama K. A. S. Conger R. D. (2004). Modeling continuity and change in family relationships with panel data, in Continuity and Change in Family Relations: Theory, Methods, and Empirical Findings, eds Conger R. D. Lorenz F. O. Wickrama K. A. S. (Mahwah, NJ: Lawrence Erlbaum), 15–62.

[B63] MacIntyre P. Gregersen T. (2012). Emotions that facilitate language learning: The positive broadening power of the imagination. Stud. Second Lang. Learn. Teach. 2, 193–213. 10.14746/ssllt.2012.2.2.4

[B64] MacIntyre P. D. Gregersen T. Mercer S. (2016). Conclusion in Positive psychology in SLA, in Positive Psychology in Second Language Acquisition, eds MacIntyre P. D. Gregersen T. Mercer S. (Bristol: Multilingual Matters), 347–379.

[B65] MacIntyre P. D. Mercer S. (2014). Introducing positive psychology to SLA. Stud. Second Lang. Learn. Teach. 4, 153–172. 10.14746/ssllt.2014.4.2.2

[B66] McCain B. (2017). Effects of teacher grit on student grit and reading achievement: A mixed-methods study [Doctoral dissertation]. Retrieved from Indiana MacIntyre, P. D., and Gregersen, T. (2012). Emotions that facilitate language learning: The positive-broadening power of the imagination. Studies in Second Language Learning and Teaching, 2(2): 193–213.

[B67] McDonald R. P. (1985). Factor Analysis and Related Methods. Hillsdale, NJ: Lawrence Erlbaum.

[B68] McDonald R. P. (1999). Test Theory: A Unified Treatment. Mahwah, NJ: Erlbaum.

[B69] Pan C. Zhang X. (2021). A longitudinal study of foreign language anxiety and enjoyment. Lang. Teach. Res. 8, 149–170. 10.1177/1362168821993341

[B70] Pavelescu L. M. Petrić B. (2018). Love and enjoyment in context: four case studies of adolescent EFL learners. Stud. Sec. Lang. Learn. Teach. 8, 73–102. 10.14746/ssllt.2018.8.1.4

[B71] Pekrun R. (2006). The control value theory of achievement emotions: assumptions, corollaries, and implications for educational research and practice. Educ. Psychol. Rev. 18, 315–341. 10.1007/s10648-006-9029-9

[B72] Piechurska-Kuciel E. (2017). L2 or L3? Foreign language enjoyment and proficiency, in Multiculturalism, Multilingualism and the Self, eds Gabryś-Barker D. Gałajda D. Wojtaszek A. Zakrajewski P. (Bristol: Multilingual Matters), 97–111.

[B73] Piña-Watson B. López B. Ojeda L. Rodriguez K. M. (2015). Cultural and cognitive predictors of academic motivation among Mexican American adolescents: caution against discounting the impact of cultural processes. J. Multicult. Couns. Devel. 43, 109–121. 10.1002/j.2161-1912.2015.00068.x

[B74] Resnik P. Dewaele J.-M. (2020). Trait emotional intelligence, anxiety and enjoyment in first and foreign language classes. Syst. Adv. 94:102324. 10.1016/j.system.2020.102324

[B75] Sudina E. Vernon T. Foster H. Del Villano H. Hernandez S. Beck D.. (2020). Development and Initial Validation of the L2-Teacher Grit Scale. TESOL Q. 55, 156–184. 10.1002/tesq.581

[B76] Teimouri Y. Plonsky L. Tabandeh F. (2020). L2 Grit: Passion and perseverance for second-language learning. Lang. Teach. Res. 107:1895. 10.1177/1362168820921895

[B77] Usher E. L. Li C. R. Butz A. R. Rojas J. P. (2019). Perseverant grit and self-efficacy: are both essential for children's academic success? J. Educ. Psychol. 111, 877–902. 10.1037/edu0000324

[B78] Vainio M. M. Daukantaite D. (2016). Grit and different aspects of well-being: direct and indirect relationships via sense of coherence and authenticity. J. Happiness Stud. 17, 2119–2147 10.1007/s10902-015-9688-7

[B79] Von Culin K. R. Tsukayama E. Duckworth A. L. (2014). Unpacking grit: motivational correlates of perseverance and passion for long-term goals. J. Posit. Psychol. 9, 306–312. 10.1080/17439760.2014.898320 31404261 PMC6688745

[B80] Wei H. Gao K. Wang W. (2019). Understanding the relationship between grit and foreign language performance among middle school students: the roles of foreign language enjoyment and classroom environment. Front. Psychol. 10:1508. 10.3389/fpsyg.2019.01508 31333541 PMC6624730

[B81] Willett J. B. Sayer A. G. (1994). Using covariance structure analysis to detect correlates and predictors of individual change over time. Psychol. Bull. 116, 363–381. 10.1037/0033-2909.116.2.363

[B82] Willett T. (1988). A cross-linguistic survey of the grammaticization of evidentiality. studies in Language. Found. Lang. 12, 51–97. 10.1075/sl.12.1.04wil

[B83] Wolters C. A. Hussain M. (2015). Investigating grit and its relations with college students' self-regulated learning and academic achievement. Metacogn. Learn. 10, 293–311. 10.1007/s11409-014-9128-9

[B84] Yamashita T. (2018). Grit and Second Language Acquisition: Can Passion and Perseverance Predict Performance in Japanese Language Learning? Unpublished MA thesis, University of Massachusetts, Amherst, MA.

[B85] Yoon S. Kim S. Kang M. (2020). Predictive power of grit, professor support for autonomy and learning engagement on perceived achievement within the context of a flipped classroom. Activ. Learn. High. Educ. 21, 233–247 10.1177/1469787418762463

